# A Phytochemical Analysis and the Pharmacological Implications of the Seagrass *Halodule uninervis*: An Overview

**DOI:** 10.3390/ph17080993

**Published:** 2024-07-27

**Authors:** Nadine Wehbe, Mikhael Bechelany, Adnan Badran, Ali Al-Sawalmih, Joelle Edward Mesmar, Elias Baydoun

**Affiliations:** 1 Department of Biology, Faculty of Arts and Sciences, American University of Beirut, Riad El Solh, Beirut 1107 2020, Lebanon; nww04@mail.aub.edu (N.W.); jm104@aub.edu.lb (J.E.M.); 2 Institut Européen des Membranes-IEM (UMR 5635), University Montpellier, CNRS, ENSCM, 34095 Montpellier, France; 3 Functional Materials Group, Gulf University for Science and Technology (GUST), Mubarak Al-Abdullah 32093, Kuwait; 4 Department of Nutrition, Faculty of Pharmacy and Medical Sciences, University of Petra, Amman 1196, Jordan; abadran@uop.edu.jo; 5 Marine Science Station, University of Jordan, Aqaba 11942, Jordan; a.sawalmih@ju.edu.jo

**Keywords:** *Halodule uninervis*, seagrass, marine ecosystem, herbal medicine, bioactive metabolites, antioxidant, antimicrobial, anticancer, antidiabetic, green nanotechnology

## Abstract

Seagrasses are marine angiosperms that inhabit tropical and subtropical regions around the world. They play a vital role in marine biodiversity and the ecosystem by providing habitats and food for several marine organisms, stabilizing sediments, and improving water quality. *Halodule uninervis* from the family *Cymodoceaceae* has been used in traditional folk medicine for the treatment of many ailments. Additionally, several identified bioactive metabolites have been shown to contribute to its pharmacological activities, including anticancer, anti-inflammatory, and antioxidant. As such, *H. uninervis* could contribute to the development of novel drugs for various diseases. This review aims to compile the phytochemical composition and pharmacological activities of *H. uninervis*. Furthermore, details about its botanical characteristics and ecological significance are also discussed. By providing valuable insights into the role of *H. uninervis* in both the marine ecosystem and biomedicine, this review helps to highlight its potential as a therapeutic agent for future drug discovery and development.

## 1. Introduction

Since ancient civilizations, traditional practitioners relied on nature for remedies to treat various ailments and promote health. In the present times, plants and other natural products continue to be utilized in modern drug development, as 80% of the world’s population relies on these natural remedies for medicinal uses [1]. Owing to their phytochemical diversity, plant extracts and their derivatives have contributed to the development of many pharmaceuticals, including drugs such as digoxin from *Digitalis lanata* for the treatment of congestive heart failure [2], morphine from *Papaver somniferum* to treat severe pain [3], paclitaxel from *Taxus brevifolia* as a chemotherapeutic agent [4,5], and quinine from *Cinchona officinalis* for the treatment of malaria [6].

Despite terrestrial plants being the main focus in drug development for centuries, marine plants have been receiving a surge of interest in recent years, as the marine environment comprises 95% of the biosphere and offers a vast reservoir of biodiversity [7]. Having to survive in extreme and dynamic environments, marine plants have evolved unique adaptation and defense strategies, which could contribute to the production of bioactive compounds with distinctive chemical structures and pharmacological properties. However, the complexity of the marine ecosystem and limited accessibility to marine plants pose a challenge in exploring their therapeutic potential [8]. So far, several marine-derived drugs from other organisms have been approved by the US Food and Drug Administration (FDA). For instance, ziconotide, a peptide discovered from the marine cone snail *Conus magus*, is used for the treatment of severe chronic pain [9]. Another approved anticancer drug is trabectedin, which is derived from the Caribbean sea squirt *Ecteinascidia turbinate* and used in the treatment of soft tissue sarcoma [10]. Seagrasses have also been used as natural remedies for the treatment of fever, muscle and stomach pains, and wounds [11]. Moreover, the use of seagrasses extended to weaving baskets, thatching roofs, crafting paper-based materials, and preparing fertilizers [12]. There are 72 species of seagrasses divided into six families, *Cymodoceaceae*, *Hydrocharitaceae*, *Posidoniaceae*, *Ruppiaceae*, *Zannichelliaceae*, and *Zosteraceae* [13]. Of particular relevance to this review, *Halodule uninervis* (Forsskål) Ascherson of the family *Cymodoceaceae* is an abundant species found in tropical and subtropical coastal regions worldwide. As it needs to thrive in extreme environments with high-stress conditions, *H. uninervis* is rich in bioactive metabolites, which act as defense mechanisms and bestow the plant with many pharmacological properties, such as anticancer, antimicrobial, and antioxidant [14,15,16].

**Figure 1:**
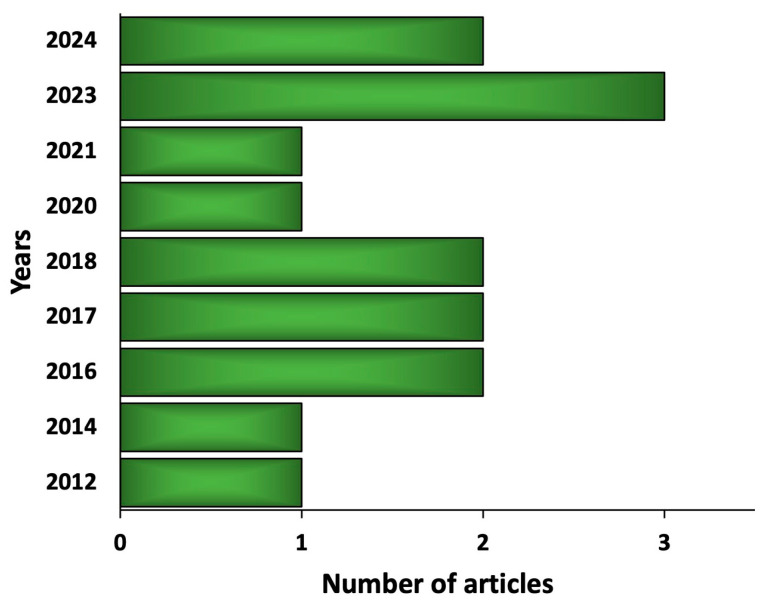
A chart showing the number of published articles on the phytochemical composition and pharmacological activities of *Halodule uninervis* from 2012 to 2024.

The therapeutic value of seagrasses was documented by several reviews, shedding light on their phytochemical composition as well as their antioxidant, anticancer, and anti-inflammatory properties, among others [17,18]. Indeed, interest in the pharmacology of seagrasses has been steadily increasing in recent years. Particularly, a PubMed search of “seagrass pharmacology” showed that related publications since 1985 have more than doubled in the last decade. Despite the noticeable increasing interest in the pharmacological activities of seagrasses, most of the published papers focus on their remarkable ecological role. Since there is a continuous need for the development of alternative therapeutics from natural sources with limited side effects, a thorough investigation of different seagrass species is required to discover new bioactive metabolites with potential medicinal use. Given the limited literature on the phytochemical composition and pharmacological properties of the seagrass *Halodule uninervis* (Figure 1), this review aims to compile all existing studies and serve as a basis for future investigations to help uncover the potential use of *H. uninervis* as a therapeutic agent. This updated review paper focuses on the phytochemical analysis and pharmacological implications of *H. uninervis*, highlighting the limitations of current studies and possible future implications. Overall, we provide (1) a description of its botanical and ecological characteristics and (2) an updated comprehensive compilation of its phytochemical composition in addition to (3) its pharmacological activities.

## 2. Taxonomic Classification of *Halodule uninervis* (WoRMS)

Seagrasses are classified into six different families, including Cymodoceaceae, Hdyrocharitaceae, Posidoniaceae, Potamogetonaceae, Ruppiaceae, and Zosteraceae. The Halodule genus belongs to the family *Cymodoceaceae*, and it comprises seven different species, including *Halodule beaudettei*, *Halodule bermudensis*, *Halodule ciliate*, *Halodule emarginata*, *Halodule pinifolia*, *Halodule uninervis*, and *Halodule wrightii*. Table 1 showcases the taxonomic classification of *Halodule uninervis* as found in the World Register of Marine Species (WoRMS).

## 3. Methods

Major scientific literature databases, including PubMed, ScienceDirect, Scopus, Chemical Abstracts, Henriette’s Herbal Homepage, and Medicinal and Aromatic Plants Abstracts, were utilized to search for and retrieve articles related to the review subject. General web searches were also conducted using Google and Google Scholar. The search period covered articles published between year 1968 and July 2024. The search used the keywords and MeSH terms for ‘*Halodule uninervis*’, AND (‘phytochemical compounds, or content, or constituents’, ‘pharmacological activities, or effects, or properties, or roles’, ‘antibacterial’, ‘anticancer’, ‘antidiabetic’, ‘antifungal’, ‘anti-inflammatory’, ‘antimicrobial’, ‘antioxidant’, or ‘clinical trials’).

## 4. Botanical and Ecological Characteristics of *Halodule uninervis*

### 4.1. Botanical Characteristics

**Figure 2:**
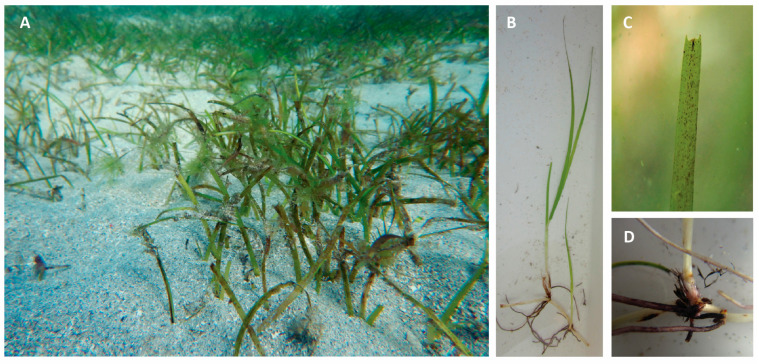
(**A**) *Halodule uninervis* in its local environment forming meadows. (**B**) *Halodule uninervis* whole plant showing the branching rhizome, roots at the nodes, short erect stems, and flat needle-like leaves. (**C**) *Halodule uninervis* leaf blade with 3 distinct points “teeth” at its tip. (**D**) *Halodule uninervis* branching rhizome and roots. Images were obtained from https://www.inaturalist.org/taxa/416176-Halodule-uninervis/browse_photos (accessed on 14 May 2024).

*H. uninervis*, commonly known as “narrowleaf seagrass” and “a’shab bahriya” in Arabic [19], is a perennial flowering plant with a vibrant green color that forms dense, large meadows under the sea resembling grassland (Figure 2A) [20].

Members of the family Cymodoceaceae are dioecious, having separate male and female flowers with the reproductive parts found on the flowering stems at the base of the leaf sheath [21]. The produced seeds have a hard seed coat and are usually released directly into the sediments, preserving them until the parent plants are destroyed [22].

*H. uninervis* spreads via a branching rhizome that roots at the nodes (Figure 2B,D). At each node, up to six very fine roots are found with a short, erect stem [21]. About two to four alternately arranged leaves emerge on each stem, with a sturdy, persistent leaf sheath up to 3.5 cm long. The leaf blades are thin, flat needle-like structures that grow straight up to 15 cm long and 0.25–5 mm wide, with three longitudinal veins and three distinct points at the leaf tip referred to as “teeth” (Figure 2C) [23,24]. Interestingly, leaf morphology changes according to the habitat type. Long, narrow leaves are found closer to the shore, whereas short, wide leaves are found in deeper areas [25]. This could be explained by the fact that plants closer to the shore would need less surface area to absorb the sunlight needed for photosynthesis.

### 4.2. Ecological Characteristics

Seagrasses are the only angiosperms to recolonize the seabed. They are categorized as halophytes and are capable of growing partially or entirely submerged under water [17]. As part of the marine ecosystem, seagrasses play a critical role in serving as habitat for different marine organisms, supporting food security, stabilizing sediments, and mitigating climate change [26,27,28].

**Figure 3:**
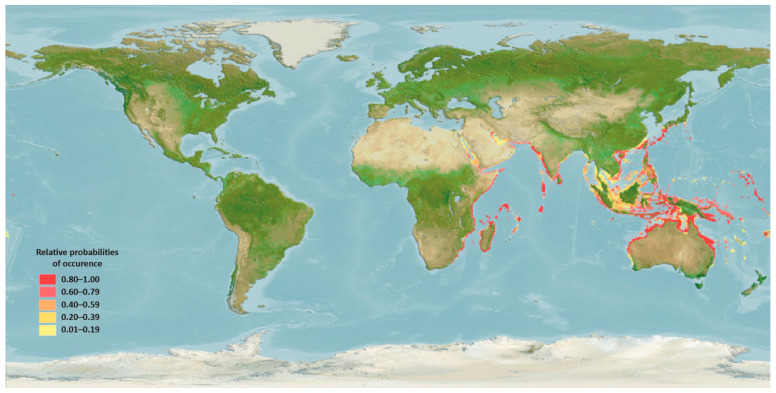
Native distribution map for *Halodule uninervis*. Map retrieved from https://www.aquamaps.org.

*H. uninervis* is a very common species that is predominantly located in tropical and subtropical regions around the world. It is widely distributed in the Indo-Pacific region [29] (Figure 3).

In the Indian Ocean, it is commonly found in areas along the coastlines of the Red Sea and the Arabian Gulf, including Egypt [30], Jordan [31], Oman [32], Qatar [33], and Saudi Arabia [34]. It also spreads from the Southeast coast of South Africa [35,36] to the islands of the western Indian Ocean, including Andaman Islands [37], Mauritius [38], and Seychelles [39]. In the Pacific Ocean, it is distributed throughout Southeast Asia, including Indonesia [40], Malaysia [41], and the Philippines [42]. It is also found in the Gulf of Thailand [43] and along the coast of Vietnam [44] and southern China [45].

*H. uninervis* grows in a variety of habitats, where it often forms dense meadows in shallow coastal waters, lagoons, exposed or sheltered coral reefs, and sandy and muddy substrates [46]. Within these habitats, it can withstand harsh environmental conditions, such as salinity fluctuations, sedimentation, temperature extremes, and wave action. In Eastern Australia, it survives the unstable and depositional environments. In the Arabian Gulf, it withstands extreme conditions of high salinity levels by regulating the concentration of salt within its tissues and maintaining osmotic balance and high temperatures [47,48]. Several adaptation strategies have evolved to help *H. uninervis* survive and persist in marine environments. For instance, its flexible leaves and anchoring rhizomes help withstand wave action and reduce the risk of damage [25,49]. The modification in the dimensions of leaves and stems also helps in obtaining sufficient sunlight, with submerged plants having longer leaves and stems compared to the ones near the shore [25].

Like other seagrasses, *H. uninervis* plays a crucial ecological role in the marine ecosystems. By forming dense meadows undersea, it provides a habitat for a myriad of marine organisms [50,51]. *H. uninervis* also helps in stabilizing sediments, reducing coastal erosion, maintaining water quality, and sequestering carbon by absorbing nutrients and trapping sediments through its root system [52,53,54]. Moreover, it contributes to the complex marine food webs by providing nourishment for herbivores.

## 5. Phytochemical Constituents of *Halodule uninervis*

### 5.1. Bioactive Metabolites

Beyond their ecological significance, seagrasses have been used in traditional folk medicine for the treatment of various ailments, including digestive disorders, inflammatory conditions, and skin diseases, among others. As it must thrive in high-stress environments, *H. uninervis* is rich in phenolic compounds that confer the plant with self-defense mechanisms. Moreover, extracts from different seagrasses have been shown to possess a variety of pharmacological activities, such as anticancer, anti-inflammatory, and antimicrobial. These properties of seagrasses are attributed to the presence of a wide array of secondary metabolites.

The preliminary phytochemical screening of *H. uninervis* revealed that it is rich in secondary metabolites, including phenols, flavonoids, quinones, tannins, terpenoids, and steroids [14,16,55,56]. However, research into the detailed phytochemical composition of *H. uninervis* remains limited. It is noteworthy to mention that the composition of bioactive metabolites in *H. uninervis* varies depending on the type of the extract. Few phenolic compounds identified in a methanolic extract of *H. uninervis* were caffeic acid, chlorogenic acid, gallic acid, and p-hydroxybenzoic acid [15]. In addition, this extract contained three flavonoids, including apigenin, apigenin-7-O-glucoside, and naringenin [15]. Moreover, it was rich in fatty acids, including linoleic acid (9Z,12Z-octadecadienoic), palmitic acid, and stearic acid [15]. Linoleic acid was also observed in an ethyl acetate extract of *H. uninervis* [16]. An investigation of an ethanolic extract of *H. uninervis* revealed the presence of 10 phenolic compounds and 9 flavonoids [57]. Some phenolic compounds included benzoic acid, caffeic acid, catechol, ellagic acid, and pyrogallol, whereas kaempferol-3-2-p-coumaroyl glucose, naringin, and quercetin were few of the identified flavonoids. In another study, the LC-MS/MS analysis of *H. uninervis* ethanolic extract revealed the presence of 4 phenolic compounds and 11 flavonoids [56]. Phenolic compounds included dihydroxybenzoic acid, hydroxybenzoic acid, vanillic acid, and coumaric acid. Identified flavonoids comprised apigenin, acacetin, diosmetin, kaempferol, quercetin glucoside, and diosmetin glucoside. A detailed list of the identified phenols and flavonoids is found in Table 2.

**Figure 4:**
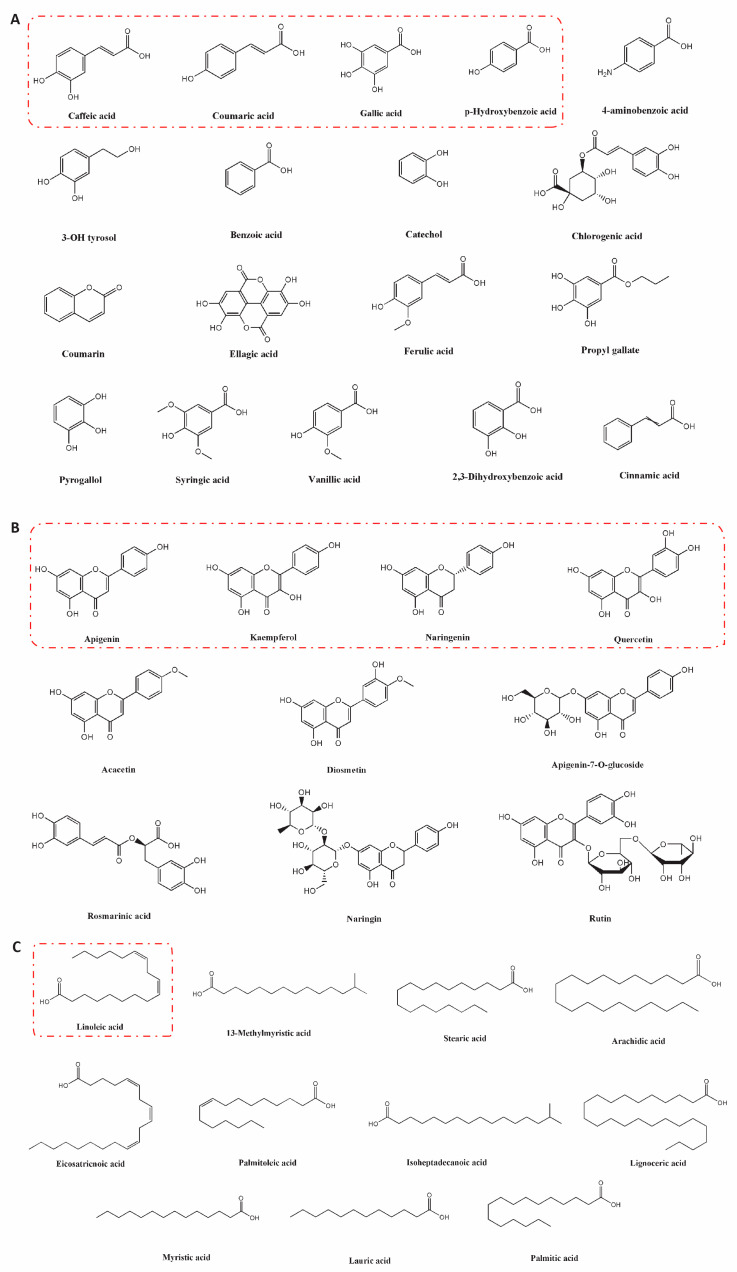
The structures of the identified (**A**) phenolic acids, (**B**) flavonoids, and (**C**) fatty acids in different extracts of *Halodule uninervis*. Red boxes highlight the major compounds in each group.

Caffeic acid, coumaric acid, gallic acid, and hydroxybenzoic acid are the major identified phenols in different extracts of *H. uninervis* (Figure 4A).

Apigenin, kaempferol, naringenin, quercetin, and their derivatives are the major identified flavonoids (Figure 4B). As evident by the aforementioned studies, compositional studies of *H. uninervis* are insufficient. Therefore, future investigations should focus on performing a detailed analysis of phenolic compounds and flavonoids found in *H. uninervis* extracts as well as other groups of bioactive metabolites.

### 5.2. Macro- and Micronutrients

Apart from the bioactive metabolites with medicinal benefits, studies have demonstrated that *H. uninervis* also contains compounds with nutritional value, including both macro- and micronutrients. Macronutrients, such as carbohydrates, lipids, and proteins, play an important role in the adaptive mechanisms against stress conditions. An analysis of an aqueous extract of *H. uninervis* showed glucose, galactose, and xylose as the major monosaccharides [15]. This extract also had high protein content, whereas an ethanolic extract from *H. uninervis* showed high protein content but had low levels of carbohydrates [57].

Other compounds with nutritional value include micronutrients, such as minerals. An analysis of essential minerals in *H. uninervis* revealed the presence of nitrogen, potassium, calcium, magnesium, and phosphorus, whereas the identified nonessential minerals were iron, zinc, manganese, and copper [58,59]. The presence of macro- and micronutrients in *Halodule uninervis* make it a valuable nutritive source for marine organisms that feed on them. Moreover, the calorific value in *H. uninervis* leaves and rhizomes was comparable to that of beetroots, carrots, ladies’ fingers, and cauliflowers [60]. Taken together, these results indicate that *H. uninervis* could contribute to human nutritional needs by being utilized in dietary and pharmaceutical supplements.

## 6. Pharmacological Activities of *Halodule uninervis*

### 6.1. Antioxidant Activities

Oxidative stress results from an imbalance between free radicals and antioxidants in the body. It is implicated in the onset and progression of many diseases, such as atherosclerosis, cancer, inflammation, and neurodegenerative disorders. Synthetic antioxidants have been extensively employed to reduce oxidative stress. However, due to concerns over the safety of these antioxidants, natural sources, such as plants, have been utilized instead, owing to the presence of bioactive metabolites that possess strong antioxidant activity. Table 3 summarizes the antioxidant potential of different *H. uninervis* extracts.

The methanolic extract of *H. uninervis* showed robust scavenging activities against the 1,1-diphenyl-2-picrylhydrazyl (DPPH) radical, indicating the presence of antioxidants [61]. Similarly, significant antioxidant activity was observed in the methanolic extract through the trolox equivalent antioxidant capacity assay, where it effectively scavenged the 2,2′-azino-bis-(3-ethylbenzothiazoline-6-sulfonic acid) radical (ABTS) [62]. The antioxidant activity of the methanol/chloroform extract, unsaponifiable matter (USM) content, and phenolic extract of *H. uninervis* was evaluated using the DPPH radical scavenging assay. The results showed that the USM content exhibited the strongest radical scavenging activity, which was attributed to the presence of dibuylhydroxy-toluene (BHT) antioxidant, a widely used preservative in food and cosmetics to prevent oxidation of free radicals [15]. An ethanolic extract of the leaves of *H. uninervis* was evaluated for its antioxidant capacity using the DPPH radical scavenging assay [56]. The findings revealed a significant antioxidant activity in a dose-dependent manner with an IC_50_ of 301.31 μg/mL.

The observed antioxidant activity of *H. uninervis* was associated with the presence of bioactive metabolites, including phenols and flavonoids, which are known antioxidant compounds that play an important role in plant defense mechanisms as well as a protective role in human health. However, the chemical assays used in the aforementioned studies are non-specific and prone to interference. Therefore, they are not enough to validate the antioxidant potential of *H. uninervis* and its associated pharmacological properties. Future research should focus on implementing in vivo methods for assessing antioxidant activities.

### 6.2. Antimicrobial Activities

Infectious diseases persist as a serious threat and a major cause of death due to the existence of antibiotic-resistant pathogens. This has urged the need to search for and develop new antimicrobial agents by utilizing natural compounds. Indeed, medicinal plants have been extensively studied as potential antimicrobial agents due to their production of bioactive compounds with known therapeutic properties. Several studies evaluated the effectiveness of *H. uninervis* extracts as antibacterial agents as illustrated in Table 4.

The results have shown that a methanolic extract from the leaves of *H. uninervis* exhibits stronger antibacterial activity against Gram-positive bacteria, such as *Bacillus subtilis* and *Listeria monocytogenes*, compared to Gram-negative bacteria, such as *Aeromonas hydrophila* and *Vibrio harveyi* [14]. Gumgumjee et al. evaluated the antimicrobial activity of *H. uninervis* leaf extracts in aqueous solution and other organic solvents [63]. The results showed that the ethanolic extract exhibited the highest level of inhibition among all solvents, whereas the aqueous extract showed no activity, with the exception of *Pseudomonas aeruginosa*. An ethanolic extract of *H. uninervis* exhibited inhibitory effects on the growth of *Bacillus cereus*, a Gram-positive bacteria, and on *Proteus vulgaris*, a Gram-negative bacteria. It also inhibited the growth of the *fungus Cryptococcus neoformas* [55]. A recent study demonstrated a potent antibacterial effect of *H. uninervis* crude extracts from leaves or roots in hexane, dichloromethane, and methanol solvents against *Salmonella typhi* [64]. The hexane extracts from both the leaves and roots had the strongest inhibitory activity.

These studies support the potential use of *H. uninervis* extracts as antibacterial agents. Further research should explore the mechanism for the observed antibacterial activity. Moreover, the effect of *H. uninervis* extracts against other pathogenic microorganisms, such as fungi, pests, and yeast, should also be investigated.

### 6.3. Larvicidal Effect

Mosquito-transmitted diseases continue to be a major cause of death worldwide. Mosquitoes act as vectors of many infectious pathogens, causing serious diseases, including dengue fever, lymphatic filariasis, malaria, Zika virus, among others. Though synthetic pesticides have been widely used to control mosquito larvae, their toxicity to non-targeted organisms and the environment necessitates the use of alternative approaches. Indeed, plant products have been utilized as natural insecticides due to their ecofriendly nature and immense potential for the control of mosquito larvae.

A study was conducted to assess the mosquito larvicidal activity of *H. uninervis* ethyl acetate extract against *Culex pipiens*, the main vector of lymphatic filariasis in Egypt. The results revealed effective inhibitory activity with a lethal concentration LC_50_ of 3.97 ppm [66]. A methanolic extract of *H. uninervis* from the Saudi Red Sea exhibited moderate toxicity against the fourth stage larvae of Aedes aegypti. The mosquito larvicidal properties of *H. uninervis* are summarized in Table 5. Overall, *H. uninervis* proves to be a promising ecofriendly agent for the control of mosquito vectors.

### 6.4. Anticancer Activities

Cancer is a major public health problem and a leading cause of death worldwide. Conventional treatment regimens for cancer are non-selective, result in adverse side effects, and grow tumor resistance. This mandated resorting to medicinal plants for alternative treatment approaches [67,68].

**Figure 5:**
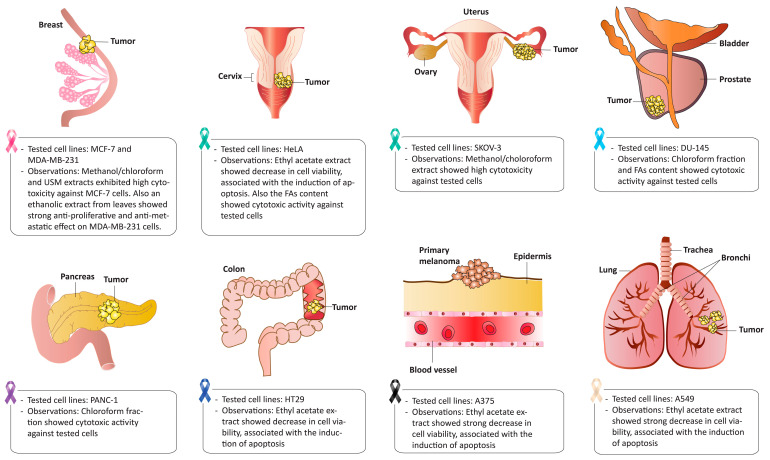
*Halodule uninervis* exhibits potent anticancer activity against several cancers.

The anticancer activity of *H. uninervis* was evaluated using an ethyl acetate extract against several cancer cell lines in vitro, including malignant melanoma A375, lung carcinoma A549, cervix adenocarcinoma HeLa, and colorectal adenocarcinoma HT29 cells. the findings indicated that the extract exhibited the highest cytotoxic activity against A549 lung cancer cells by inducing apoptosis [16]. In another study, different seagrasses collected from the Saudi Red Sea were screened for their cytotoxic activity against several cancer cell lines, including breast cancer MCF-7, prostate cancer DU-145, HeLa, ovarian cancer SKOV-3, and pancreatic cancer PANC-1 [15]. The crude extract of *H. uninervis* showed a potent cytotoxicity against SKOV-3 cells and demonstrated the most significant inhibitory effects on MCF-7 cells compared to other treatments. The chloroform fraction of the extract inhibited the viability of DU-145 and PANC-1 cells. Additionally, the extracted free fatty acids (FAs) showed anti-proliferative activity against and HeLa cells, while unsaponifiable matter (USM) exhibited cytotoxic effects only against MCF-7 cells. The anticancer activity of a crude ethanolic extract of *H. uninervis* leaves was investigated against triple-negative breast cancer MDA-MB-231 cells [56]. Results demonstrated a dose- and time-dependent decrease in cell proliferation through the induction of G_0_/G_1_ cell cycle arrest and the activation of the intrinsic apoptosis pathway. Moreover, the extract mitigated cancer metastasis by inhibiting cell adhesion, migration, invasion, and in ovo angiogenesis. These effects of *H. uninervis* were potentially mediated by the downregulation of the proto-oncogenic STAT3 signaling pathway. The promising anticancer activities of *H. uninervis* are illustrated in Figure 5 and summarized in Table 6.

However, most of the cited studies used cell viability assays to evaluate the anticancer activity of different *H. uninervis* extracts. This assay works best for screening purposes; therefore, additional research is required to confirm the anticancer potential of *H. uninervis* and to investigate the underlying molecular mechanisms.

### 6.5. Antidiabetic Effect

Diabetes is a chronic metabolic disorder characterized by hyperglycemia, elevated blood glucose concentration, due to insulin deficiency or malfunction. Uncontrolled diabetes leads to serious complications in many organs, including a loss of vision and kidney function, heart attacks, and strokes. The use of herbal medicine for the treatment of diabetes has been favored over synthetic drugs due to its low cost and low incidence of side effects.

The antidiabetic potential of *H. uninervis* extracts using different extraction solvents was tested in vitro and in vivo (Table 7).

**Figure 6:**
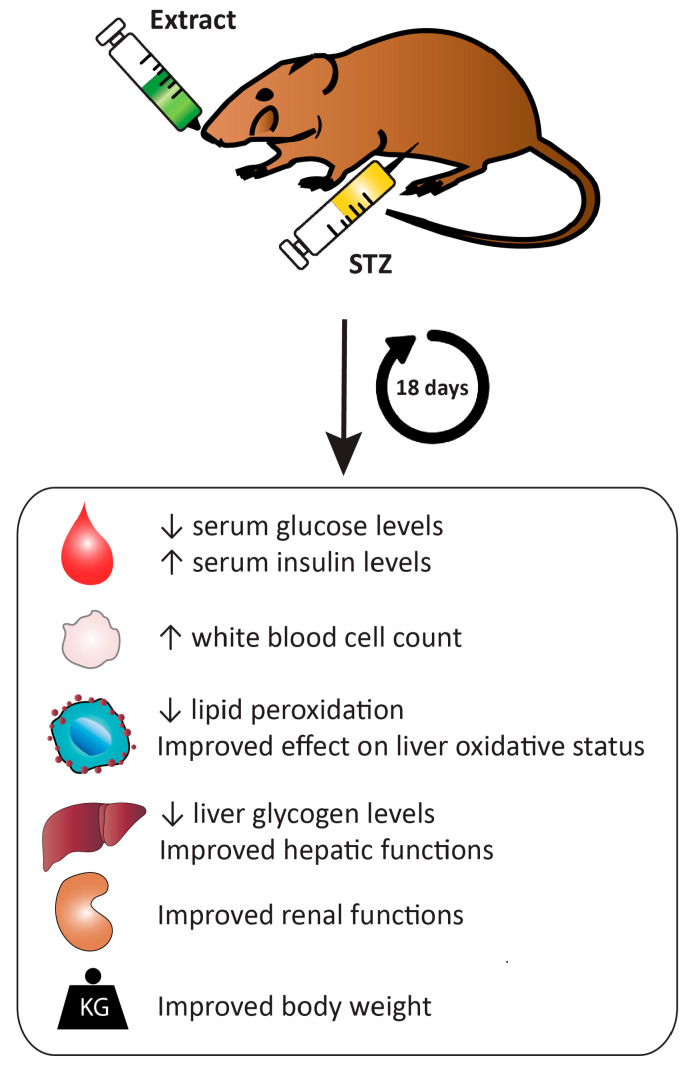
*Halodule uninervis* has an antidiabetic effect. The oral administration of a methanolic extract of *H. uninervis* in streptozotocin-induced diabetic mouse model improved the glycemic profile.

A recently conducted study evaluated the antidiabetic activity of *H. uninervis* extract with ethanol and ethyl acetate solvents [69]. The in vitro inhibition of the α-glucosidase enzyme assay revealed that the ethanolic extract possesses better antidiabetic activity compared to ethyl acetate extract. In a Streptozotocin-induced diabetic mouse model, the oral administration of methanolic extract from *H. uninervis* significantly reduced serum glucose levels [70]. Additionally, the extract recovered weight loss observed in diabetic mice, improved low white blood cell count, and exhibited a protective effect on liver oxidative status. This is presented in Figure 6 and Table 7. These results verify the potential use of *Halodule uninervis* as a potential anti-hyperglycemic agent for the treatment of diabetes.

### 6.6. Green Nanotechnology

Nanotechnology is a multidisciplinary field that involves the design, production, and characterization of materials at the nanoscale. At this scale, materials exhibit distinctive properties, rendering them highly valuable for implications across several sectors, including agriculture, cosmetology, electronics, food industry, and medicine [71]. Nanomaterials can be broadly classified into carbon-based, composite, organic, and inorganic based on their structural composition [72]. Silver nanoparticles (AgNPs), a type of metal-based inorganic nanomaterials, exhibit a high surface area-to-volume ratio and the ability to release silver ions, making them potent antimicrobial agents [73]. They have been integrated into dental products, medical implants, surgical tools, wound dressings, and textiles to prevent infections and promote healing. Conventional methods for synthesizing AgNPs include chemical processes through reduction reactions [74,75], physical methods such as laser ablation [76,77], and green synthesis routes via the use of biomaterials such as bacteria [78], fungi [79], and plant extracts [80]. The green synthesis of AgNPs ensures the production of ecofriendly and nontoxic nanoparticles, with enhanced stability, biocompatibility, and biological activities.

**Figure 7:**
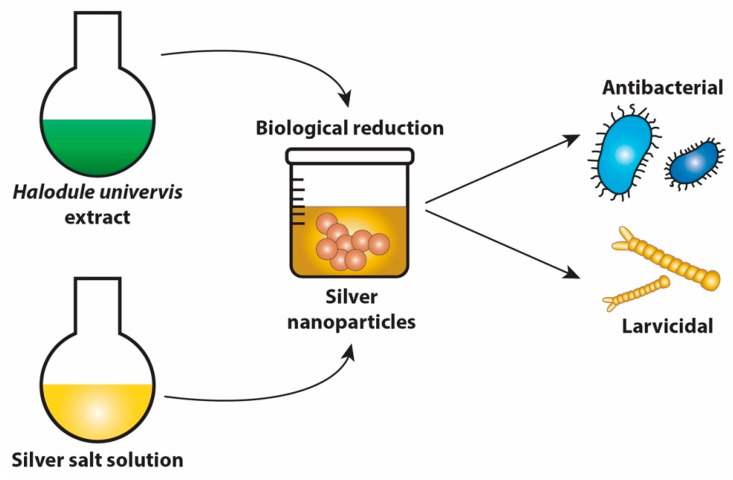
*Halodule uninervis* is used in the green synthesis of silver nanoparticles (AgNPs) with antibacterial and larvicidal activities.

*H. uninervis* aqueous extract has been utilized as a reducing and capping agent for the green synthesis of silver nanoparticles (AgNPs). The antibacterial activity of *H. uninervis*-synthesized AgNPs was evaluated against *Bacillus subtilis*, *Klebsiella pneumoniae*, and *Salmonella typhi*, with the strongest inhibitory activity observed against *B. subtilis* [65] (Table 4). Additionally, the synthesized AgNPs showed significant mosquito larvicidal activity (Table 5). Their small size allows them to pass through the insect cuticle and induce changes in cell morphology and physiological processes [65]. Figure 7 illustrates the antibacterial and larvicidal activities of *H. uninervis*-synthesized AgNPs. Overall, these results underscore the potential of *H. uninervis* in the green synthesis of nanoparticles, warranting further investigation, particularly due to their potent activity even at low doses.

## 7. Safety Profile

As the use of herbal medicinal products is globally increasing, major concerns surrounding their safety profile are emerging. Since most of these products are classified as foods or dietary supplements in most countries, the evaluation of their safety or toxicity is not mandatory prior to market release [81,82,83]. However, to avoid potential adverse effects to human health, toxicological studies are essential when developing herbal medicine to ensure its efficacy, quality, and safety. Many studies evaluated the safety profile of extracts from different seagrasses, and they were found nontoxic with a potential use in pre-clinical trials [64,84,85,86,87].

Toxicological studies for *H. uninervis* extracts are still limited. Acute toxicity studies of the methanolic *H. uninervis* extract showed no mortality or toxic effects in mice administered intraperitoneally at a dose range of 50 to 450 mg/kg. Animals were kept under observation to identify symptoms of toxicity for two weeks following treatment [70]. The safety of different extracts of *H. uninervis* leaves and roots was determined by the brine shrimp lethality assay. Brine shrimp eggs were treated with a range of 24 to 240 mg/mL of *H. uninervis* extracts for 24 h. The results revealed that both hexane and dichloromethane extracts were nontoxic with no significant difference between them or between the leaves and roots [64]. Overall, further toxicological evaluation is necessary to validate the safety profile of *H. uninervis* for therapeutic purposes.

## 8. Conclusions and Future Implications

Plant-derived natural products have been extensively used in drug discovery and development for centuries, providing a diverse array of bioactive metabolites with therapeutic potentials. Compared to terrestrial plants, research on the medicinal properties of marine plants is relatively limited. *Halodule uninervis* is a marine angiosperm that plays a crucial role in coastal marine ecosystems, yet scientific research on its therapeutic potential is still scarce. Based on its available phytochemical profile, *H. uninervis* contains various bioactive metabolites known for their antioxidant, anticancer, antimicrobial, and anti-inflammatory properties. Therefore, as the demand for natural and alternative therapeutics grows, the use of *H. uninervis* in novel drug development and therapy is potentially promising. Nonetheless, further investigation is required to thoroughly analyze the phytochemical composition of *H. uninervis* and evaluate the variation of bioactive metabolites found in extracts from different parts of the seagrass. Future studies should also aim to identify the bioactive metabolites mediating the biological activities of *H. uninervis*. Isolating and characterizing new metabolites from *H. uninervis* could reveal novel molecular mechanisms of action that could be utilized for the development of drugs targeting many diseases. These bioactive metabolites could also be used in nutraceuticals and the food industry. Furthermore, after identifying and isolating the bioactive metabolites, the safety profile and efficacy of these compounds should be evaluated in vivo models before being tested in clinical trials and used for drug development. It is important to note that there is not enough evidence in the literature highlighting the anti-inflammatory activity of *H. uninervis*. As such, future studies should focus on investigating its anti-inflammatory potential since chronic inflammation is the onset of various diseases, including atherosclerosis, cardiovascular, diabetes, and cancer. Despite the limited knowledge present about its phytochemical profile and pharmacological activities, *H. uninervis* holds a promising potential in the pharmaceutical industry since its use, among other seagrasses, as natural remedies in traditional medicine has been well documented. Unfortunately, *H. uninervis* faces various threats, including habitat loss and degradation due to coastal development, pollution, sedimentation, and climate changes [88]. Conservation efforts, such as strengthening scientific research on seagrass meadows, managing pollutant emissions, and launching awareness campaigns, should be implemented to preserve this valuable marine species [89]. Overall, compounds derived from *H. uninervis* and other seagrasses may provide useful leads for the development of novel pharmaceutical drugs.

## Figures and Tables

**Table 1:** Taxonomic classification of *Halodule uninervis*.

Kingdom	Plantae
Phylum	Tracheophyta
Class	Magnoliopsida
Order	Alismatales
Family	Cymodoceaceae
Genus	*Halodule*
Species	*Halodule uninervis*
Binomial name	*Halodule uninervis* (Forsskål) Ascherson

**Table 2:** Bioactive metabolites in *Halodule uninervis*.

Extract	Analytical Methods	Main Results	Compounds	Reference
Methanolic extract of the leaves	HPLC	8 phenolic compounds, 3 flavonoids, and 12 fatty acids	- Phenolic compounds: caffeic acid, chlorogenic acid, cinnamic acid, *p*-coumaric acid, gallic acid, *p*-hydroxybenzoic acid, propyl gallate, and syringic acid. - Flavonoids: apigenin, apigenin-7-O-glucoside, and naringenin. - Fatty acids: 13-Methylmyristic acid, α-Linoleic acid, Arachidic acid, Eicosatrienoic acid, Isoheptadecanoic acid, Lauric acid, Lignoceric acid, Linoleic acid, Myristic acid, Palmitic acid, Palmitoleic acid, and Stearic acid	[15]
Ethanolic extract of the root and shoot parts	HPLC	10 phenolic compounds and 9 flavonoids	- Phenolic compounds: 4-aminobenzoic acid, 3-OH tyrosol, benzoic acid, caffeic acid, catechol, coumarin, ellagic acid, ferulic acid, gallic acid, and pyrogallol - Flavonoids: apigenin, apigenin 7-glucose, kaempferol, kaempferol-coumaroyl glucoside, naringin, naringenin, quercetin, rosmarinic acid, rutin.	[57]
Ethanolic extract of the leaves	LC-MS/MS	4 phenolic compounds and 12 flavonoids	- Phenolic compounds: dihydroxybenzoic acid, *p* -hydroxybenzoic acid, vanillic acid, and *p*-coumaric acid. - Flavonoids: acacetin, apigenin, apigenin glucoside, diosmetin, diosmetin glucoside, kaempferol, kaempferol coumaroyl-glucoside, kaempferol glucoside, kaempferol pentoside, quercetin coumaroyl-glucoside, quercetin glucoside, and quercetin pentoside.	[56]

**Table 3:** The antioxidant effects of *Halodule uninervis*.

Extract	Dose	Methods	Observations	References
Methanolic extract	500, 1000, 1500, and 2000 ppm	DPPH radical scavenging assay and Ferric reducing power (FRAP)	- Showed strong antioxidant activity. - IC_50_ 1575 ppm.	[61]
Methanolic extract	N/A	FRAP and Trolox equivalent antioxidant capacity (TEAC) assays	- Showed strong antioxidant activity.	[62]
Methanol/chloroform extract, USM ^1^ content, phenolic extract	100–1000 mg/mL	DPPH radical scavenging assay	- Mild antioxidant activity. - USM content showed the strongest activity with IC_50_ 4.1 ± 1.98 mg/mL.	[15]
Ethanolic extract of the leaves	5, 10, 25, 50, 100, 200, and 400 μg/mL	DPPH radical scavenging assay	- Significant antioxidant activity. - IC_50_ 301.31 μg/mL.	[56]

^1^ Unsaponifiable matter.

**Table 4:** The antimicrobial activities of *Halodule uninervis*.

Extract	Dose	Experimental Model	Main Results	Reference
Methanolic extract of the leaves	500, 1000, 1500, and 2000 ppm	- Method: Agar disk diffusion - Microorganisms: *Aeromonas hydrophila*, *Vibrio harveyi*, *Bacillus subtilis*, *Listeria monocytogenes*	- Good antibacterial activity. - More effective against Gram-positive bacteria.	[14]
Aqueous extract of the leaves	N/A	- Method: Agar disk diffusion - Microorganisms: *B. subtilis,* Methicillin-Resistant *Staphylococcus aureus* (MRSA), *Staphylococcus aureus*, *Micrococcus luteus*, *Escherichia coli*, *Klebsiella pneumoniae, Pseudomonas aeruginosa*	- Good activity only against *P. aeruginosa*.	[63]
Extract of the leaves in organic solvents (chloroform, ethanol, ethyl acetate, petroleum ether)	N/A	- Method: Agar disk diffusion - Microorganisms: *B. subtilis*, *S. aureus*, *M. luteus*, *E. coli*, *K. pneumoniae*, *P. aeruginosa*	- Ethanolic extract exhibited the strongest antibacterial activity. - Highest level of inhibition against *P. aeruginosa*.	[63]
Ethanolic extract	10 mg/mL	- Method: Agar disk diffusion - Microorganisms: *Micrococcus sp.*, *Bacillus cereus*, *Enterococcus faecalis*, *Proteus vulgaris*, *P. aeruginosa*, *Enterobacter cloacae*, *Grotricum candidum*, *Syncephalastrum racemosum*, *Penicillium marnefeii*, *Cryptococcus neoformas*	- Good activity against *B. cereus* Gram-positive bacteria. - Good activity against *P. vulgaris* Gram-negative bacteria. - Good activity against *C. neoformas* fungi.	[55]
Extract of the leaves or roots in methanol, dichloromethane, and hexane	100 mg/mL	- Method: Agar disk diffusion - Microorganisms: *Salmonella typhi*	- Strong antimicrobial activity. - Hexane extract (leaves and roots) showed the highest activity. - Root extract in dichloromethane showed an inhibitory activity. - Leaf extract in methanol exhibited antibacterial activity.	[64]
*H. uninervis*-synthesized AgNPs ^1^	25, 50, 100 ppm	- Method: Agar disk diffusion - Microorganisms: *B. subtilis*, *K. pneumoniae*, *S. typhi*	- Good antibacterial properties. - Strongest inhibitory activity was against *B. subtilis*.	[65]

^1^ Silver nanoparticles.

**Table 5:** The larvicidal effect of *Halodule uninervis*.

Extract	Dose	Methods	Main Results	Reference
Ethyl acetate extract	0–8 ppm	- Method: Standard WHO method - Organisms: *Culex pipiens*	- Had effective mosquito larvicidal activity. - LC_50_ 3.97 ppm.	[66]
Methanolic extract	50, 100, 300, 500, 700 ppm	- Method: Larvicidal toxicity - Organisms: *Aedes aegypti*	- Moderate toxicity. - LC_50_ 295.629 ppm.	[65]
*H. uninervis*-synthesized AgNPs ^1^	5, 10, 15, 20, 25 ppm	- Method: Larvicidal toxicity - Organisms: *Aedes aegypti*	- Exhibited high toxicity. - LC_50_ 12.455 ppm. - Observed morphological deformities: shrinkage of internal cuticle, swelling of apical cells, and pigmentation.	[65]

^1^ Silver nanoparticles.

**Table 6:** The anticancer activities of *Halodule uninervis*.

Extract	Dose	Experimental Model	Observations	References
Ethyl acetate extract	25, 50, and 100 mg/mL	- Method: MTT ^1^ assay, AO/EtBr staining, DNA fragmentation assay - Cells: A375, A549, HeLa, HT29.	- Concentration-dependent decrease in cell viability against all tested cell lines. - Highest activity against A549 cell line via the induction of apoptosis. - Treated cells exhibited morphological changes including shrinkage, distortion, and rounded.	[16]
Methanol/chloroform extract	100–1000 mg/mL	- Method: SRB ^2^ assay. - Cells: MCF-7, DU-145, HeLa, SKOV-3), PANC-1.	- Only the crude extract showed a potent cytotoxicity against SKOV-3 cell (IC_50_ 0.52 mg/mL) - Crude extract exhibited the highest cytotoxic activity against MCF-7 cells (IC_50_ 2.30 mg/mL) among other treatments.	[15]
Chloroform fraction	100–1000 mg/mL	- Method: SRB assay. - Cells: MCF-7, DU-145, HeLa, SKOV-3), PANC-1.	- Activity against DU-145 and PANC-1 cells.	[15]
FAs content	100–1000 mg/mL	- Method: SRB assay. - Cells: MCF-7, DU-145, HeLa, SKOV-3), PANC-1.	- Activity against DU-145 and HeLa cells.	[15]
USM ^3^ content	100–1000 mg/mL	- Method: SRB assay. - Cells: MCF-7, DU-145, HeLa, SKOV-3), PANC-1.	- Activity only against MCF-7 cells.	[15]
Ethanolic extract of the leaves	100 and 200 μg/mL	- Method: proliferation assays (MTT and cell cycle analysis), cancer phenotype assay (migration, invasion, aggregation, and adhesion), and in ovo assay. - Cells: MDA-MB-231.	- Dose- and time-dependent decrease in proliferation by inducing cell cycle arrest and activating apoptosis. - Attenuation of cancer metastasis by inhibiting cell migration, invasion, adhesion, aggregation, and angiogenesis. - Downregulation of STAT3 signaling pathway.	[56]

^1^ 3-(4,5-dimethylthiazol-2-yl)-2,5-diphenyltetrazolium bromide; ^2^ Sulforhodamine B; ^3^ Unsaponifiable matter.

**Table 7:** The antidiabetic effects of *Halodule uninervis*.

Extract	Dose	Experimental Model	Observations	References
Ethanolic extract	125, 250, 500, 1000, and 2000 ppm	In vitro inhibition of α-glucosidase enzyme assay	- IC_50_ is 74.99 ppm	[69]
Ethyl acetate extract	125, 250, 500, 1000, and 2000 ppm	In vitro inhibition of α-glucosidase enzyme assay	- IC_50_ is 1517.05 ppm	[69]
Methanolic extract	150 and 250 mg/kg	Streptozotocin-induced diabetic mouse model	- Dose-dependent decrease in serum glucose levels. - Recovered weight loss and low white blood cell count. - Enhanced liver and kidney functions.	[70]
